# Anorexia Nervosa and Osteoporosis: A Possible Complication to Remember

**DOI:** 10.7759/cureus.52670

**Published:** 2024-01-21

**Authors:** André Rosas Pereira, Marta Costa, Gustavo G Costa, Ana Sofia Carvalho

**Affiliations:** 1 Family Medicine, USF Cidade Jardim, Viseu Dão Lafões, PRT; 2 Internal Medicine, Centro Hospitalar Tondela-Viseu, Viseu Dão Lafões, PRT; 3 Family Medicine, UCSP Fornos de Algodres, Guarda, PRT

**Keywords:** back pain, feeding and eating disorders, spinal fractures, osteoporosis, anorexia nervosa

## Abstract

Anorexia nervosa (AN) belongs to the spectrum of food disorders and affects approximately 2.9 million people worldwide. It is responsible for numerous and serious medical complications. Osteoporosis is a common complication, and the decrease in bone mineral density (BMD) is one of the few potentially irreversible consequences of AN. When associated with AN, it can manifest at a very young age, possibly leading to irreparable damage. We describe the case of a 30-year-old woman with a one-year evolution diagnosis of AN, complaining of back pain. Physical examination revealed a slight elevation of the right shoulder and pain at compression of paravertebral right dorsal musculature with a palpable strained muscle. Full-length X-ray imaging of the dorsal spine revealed a slight dextroconvex dorsolumbar scoliosis. A dorsal spine computerized tomography (CT) was performed, confirming a fracture of the upper platform of the sixth dorsal vertebrae. Osteodensitometry showed lumbar spine osteoporosis and femoral osteopenia. The decrease in BMD and, later on, the development of osteoporosis can occur in both types of AN. It is a severe complication that affects up to 50% of these patients. It can be irreversible and increase the lifetime risk of bone fractures and, therefore, morbimortality. Low body weight and body mass index (BMI) strongly correlate with the decrease in BMD. Treatment of osteoporosis associated with AN is not standardized and clearly labeled. Weight gain is described as the strategy with the most impact in reversing the loss of bone mass and increasing the BMD. The regularization of gonadal function also seems to independently potentiate the increase of BMD. The occurrence of long bone and vertebrae fractures frequently results in a decrease in height and chronic back pain, culminating in greater morbimortality and healthcare costs. This clinical case aims to show theclose relationship between restrictive food disorders and the decrease of BMD and the subsequent development of osteoporosis and its complications. Although rare in young and healthy people, when associated with restrictive food disorders, it should raise a red flag in its clinical evaluation. Preventing osteoporosis development and reduction of fracture risk in this population is essential. The current absence of consistent evidence regarding screening of osteoporosis in this particular group should raise awareness and promote further larger-scale studies to establish standardized recommendations concerning not only screening but also pharmacological treatment of osteoporosis in patients with AN.

## Introduction

Anorexia nervosa (AN) belongs to the spectrum of food disorders and is characterized by an intense or pathological fear of gaining weight associated with a distorted self-perception of one’s body figure, resulting in practices of exaggerated caloric restriction and, therefore, low body weight [1,2]. It can be divided into two subtypes: restricting type (AN-R) and binge-eating/purging type (AN-BP). This psychiatric disorder affects approximately 2.9 million people worldwide and has a yearly incidence of eight per 100,000 [1].

The reduction of energy intake, inherent to all subtypes of AN, is responsible for a central dysregulation in energetic homeostatic control and diversity of metabolic changes that culminate in complications across almost all of the bodily organic systems [2], namely, cardiovascular (i.e., hypotension, bradycardia, and prolonging of QT interval), hydro electrolytic (i.e., hyponatremia and hypokalemia), metabolic (i.e., hypoglycemia and hypoalbuminemia), bone metabolism (i.e., osteopenia and osteoporosis), gastrointestinal, neurological, and hematological [2].

AN is responsible for innumerous and serious medical complications with the highest mortality rate of all psychiatric disorders [2]. Although some of these medical complications are well-established and easily associated with AN, many can still be hard to detect and, therefore, must be prevented [2].

Osteoporosis is a common complication [3], and the decrease in bone mineral density (BMD) is one of the few potentially irreversible consequences of AN [2,3]. It is defined by a T-score under -2.5 (values between -1.5 and -2.5 translate osteopenia) obtained through osteodensitometry [3]. When associated with AN, it can manifest at a very young age possibly, leading to irreparable damage even when nutritional intake and body weight are restored to normal values [3]. Although already described in the literature, this association is often detected at late or advanced stages with chronic and severe consequences.

## Case presentation

We describe the case of a 30-year-old woman with a one-year evolution diagnosis of AN, a history of restrictive behavior for the past three years, with a current body mass index (BMI) of 17.7 kg/m^2^ and a chronic mild anemia associated with dietary intake restriction. The patient was medicated with an oral iron supplement and hormonal contraceptive containing cyproterone acetate (2 mg) and ethinylestradiol (0.035 mg). She practiced regular physical activity and had no toxicological habits or other relevant priors to report. Regarding family background, there was a healthy identical twin sister and a mother with a history of thyroid cancer.

The patient presented to her primary care doctor complaining of back pain with two months in duration and progressively worsening. Trauma history was denied, but she mentioned a mild physical strain as a potential trigger. She was already evaluated in the urgent care service, performed a dorsal spine x-ray with no signs of fracture, and treated with an oral nonsteroidal anti-inflammatory (acemetacin 60 mg taken twice daily) for seven days, with no improvement. Ever since this episode the patient had localized pain exacerbated mainly by upper body bilateral rotation and physical exertion, limiting her sports practice and daily activities. There was no complaint of pain while at rest or other symptoms suggestive of radiculopathies, such as paresthesia, muscle strength decrease, or impairment.

Physical examination revealed a slight elevation of the right shoulder and pain at compression of paravertebral right dorsal musculature with a palpable strained muscle. No pain was felt at the compression of the dorsal spinal or transverse apophysis, and no signs of neurological deficits were identified.

**Figure 1:**
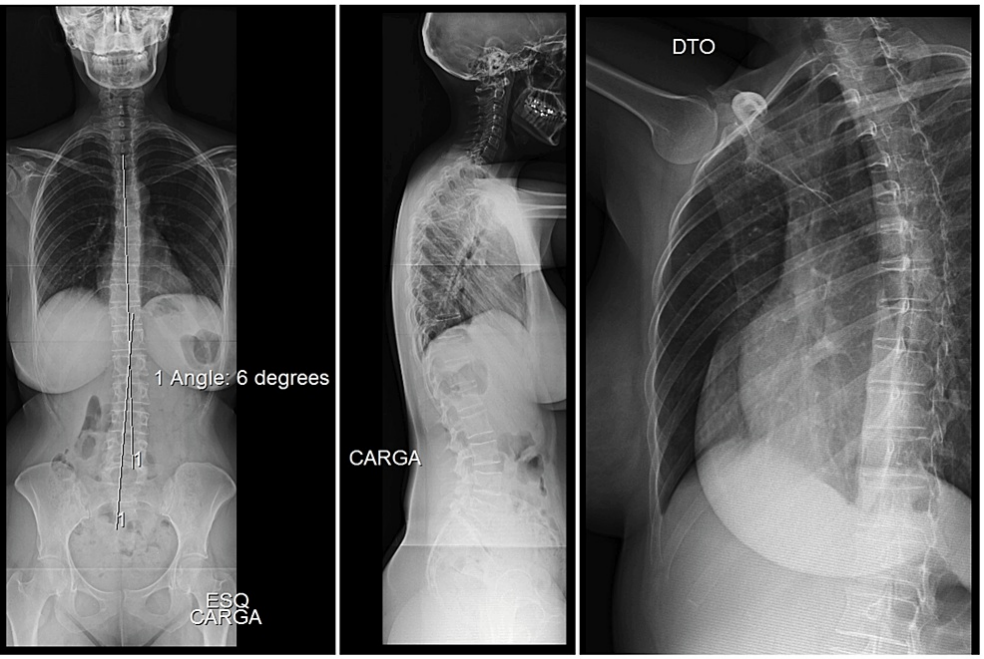
Full-length spine x-ray (antero-posterior and lateral incidence) and costal grid x-ray - from left to right Full-length spine x-ray (antero-posterior and lateral incidence) showing a sinister convex lumbar scoliosis with a six-degree angle and a subtle dextro convex dorsolumbar scoliotic attitude. Costal grid x-ray with no relevant findings.

**Figure 2:**
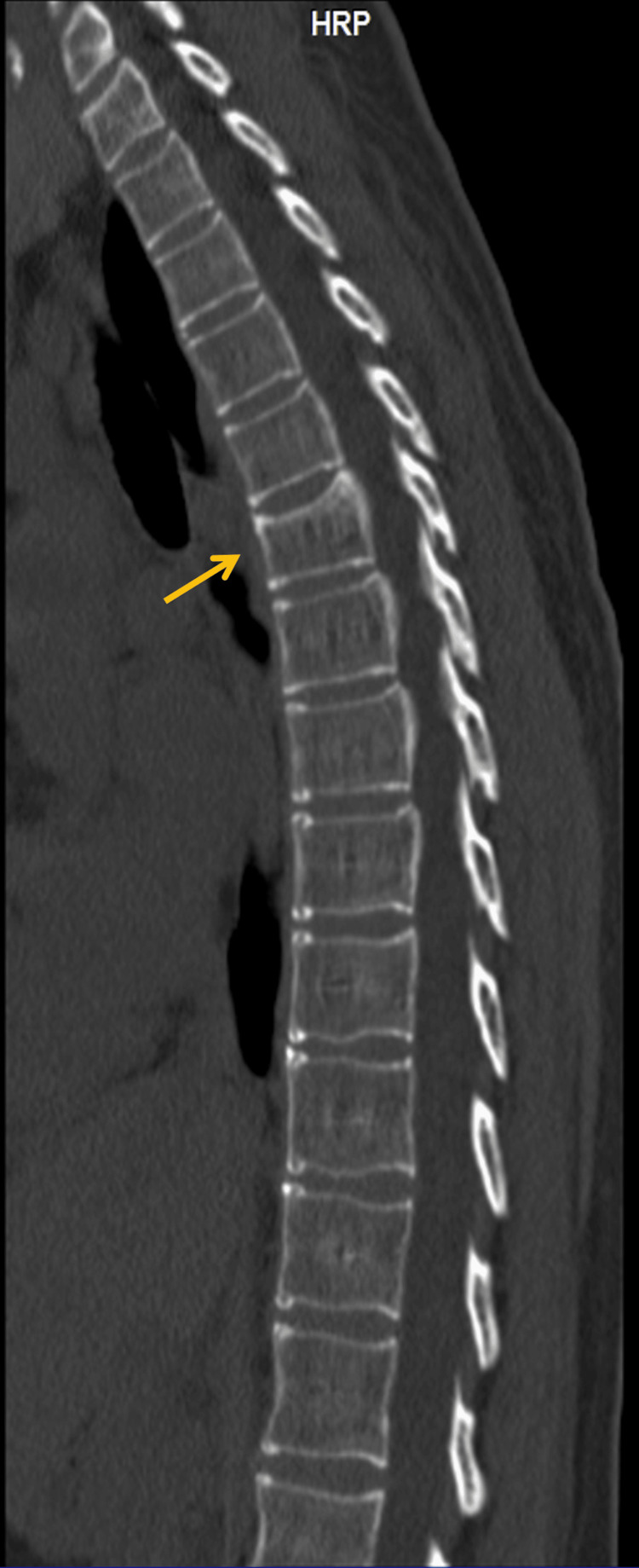
Dorsal spine CT scan Dorsal spine CT scan revealing a fracture of the upper platform of the sixth dorsal vertebrae (yellow arrow) with slight depression and associated subchondral sclerosis with very slight flattening of the vertebrae that remained aligned, without compromising the vertebral canal or the contiguous intervertebral foramina. Disc herniations were not identified, and the vertebral canal and intervertebral foramina had adequate dimensions.

A full-length spine and costal grid x-ray was requested, and she was treated with an oral muscle relaxant (thiocolchicoside 4 mg taken twice daily), paracetamol (1,000 mg taken up to three times daily), and an anti-inflammatory patch (flurbiprofen 40 mg applied once every 12 hours) for about two weeks. The costal grid x-ray presented no alterations, but the full-length spine x-ray showed a slight dextroconvex dorsolumbar scoliosis (Figure 1). There was no improvement despite pharmacological treatment and three weeks of physical therapy. Given the clinical outcome, a dorsal spine computerized tomography (CT) was performed, revealing a fracture of the upper platform of the sixth dorsal vertebrae (Figure 2). The patient was immediately referred to an orthopedics consultation where a conservative approach was advised.

Further investigation with osteodensitometry revealed a lumbar spine T-score of -3.0 and a femoral neck T-score of -1.6, confirming the diagnosis of lumbar spine osteoporosis and femoral osteopenia. Blood workup showed a moderate vitamin D deficiency (14.7 mg/mL), with normal levels of calcium, phosphate, and parathyroid hormone.

The patient was prescribed vitamin D supplementation, started treatment with alendronic acid, and kept being followed for pain management. She was also referred to further physical therapy and sent to endocrinology evaluation.

## Discussion

The decrease in BMD and later on the development of osteoporosis in patients with AN is a complication that affects up to 50% of these patients [3,4]. It can occur in both AN subtypes [3], although some evidence suggests a higher prevalence of osteoporosis in AN-R and osteopenia in AN-BP [5]. Unlike most complications that develop over the course of AN that can be reversible with weight gain and nutritional rehabilitation, this one might be irreversible and increase the lifetime risk of bone fractures, specifically vertebrae compression and long bone fractures, therefore increasing morbimortality [2,3].

The loss of BMD seems to initiate rapidly shortly after the onset of AN [3,4]. Low body weight and BMI, characteristics of AN, strongly correlate with the decrease in BMD, translating into an increased risk of developing osteoporosis [1,5]. These seem to be strong predictors of bone loss and risk of fractures [1]. Additionally, BMD is inversely related to the duration of time that the patient has a low BMI [5]. Patients with AN-R seem to have lower BMI compared to AN-BP, which could explain the higher prevalence of osteoporosis in the first subtype [5].

As AN is more prevalent in females, evidence surrounding AN-related bone diseases is most frequently described in the female gender [5]. A decreased gonadal function, commonly observed in women with AN, is also a strong predictor of osteoporosis risk and resulting fractures [1]. Other hormonal and metabolic disturbances observed in patients with AN, such as growth hormone resistance, low levels of leptin, and hypocortisolemia, have been associated and seem to correlate with the loss of bone mass [1,4], though the evidence is still inconsistent [1,5]. Estrogen deficiency seems to have a main role in the development of osteoporosis in women with AN [1,2,4]. Up to 70% of women with AN present amenorrhea or other menstrual irregularities, which frequently is correlated with low BMD when compared with eumenorrheic women, suggesting that the latter are protected from this bone mass loss [1,2,4,5]. The inhibitory effect of estrogens on osteoclasts, decreasing bone reabsorption, could explain the greater risk of developing osteoporosis in amenorrheic women [1,4,5]. Despite this, low BMD is reported at similar rates in adult and adolescent males [5]. In men, AN is associated with a decrease in testosterone levels [1-3], correlating with lower BMD [5].

Children and adolescents are a group of particular risk due to the fact that they are in a growth and development stage with the peak bone mass yet to be achieved [1,2]. Disruption or interference in this process of attaining peak bone mass results in an increased risk of osteoporosis, future fractures, and possible irreversibility of the related medical consequences [1,2,5]. It seems that there is not a statistically significant association between gender in adolescents with AN, either for cut-off weight values or for BMI [2]; therefore, the total amount of weight loss seems to play a more relevant role in the decrease of BMD [2].

Regarding methods of BMD measurement, in the general population, low BMD measured through dual-energy x-ray absorptiometry (DEXA) is a well-proven predictor of fracture risk, but, in AN patients, the evidence is not conclusive, with some studies suggesting that other methods should be applied [5]. The alternatives might include high-resolution peripheral quantitative computerized tomography and bone marrow adiposity measurement, although larger-scale studies are needed to establish correlations with fracture risk and verify its clinical use [5].

Treatment in osteoporosis associated with AN is not standardized and clearly labeled, especially concerning pharmacological treatment, as opposed to osteoporosis in post-menopausal women. Weight gain is described as the strategy with the most impact in reversing the loss of bone mass and increasing the BMD [1,3,5]. The regularization of gonadal function also seems to independently potentiate the increase of BMD [1,3-5].

The pharmacological treatment with bisphosphonates, teriparatide, or insulin-like growth factor 1 (IGF-1) supplementation may be considered; however, the available information regarding the long-term efficacy and safety of these therapeutic agents in this entity is still insufficient [1,3,4]. The role of denosumab is still not formally validated, and it may require further studies [1,5]. Although the role of bisphosphonates in patients with AN is still not entirely explained, its use seems to reduce the risk of future vertebrae fractures by up to 70% and up to 50% of hip fractures [3]. Adequate vitamin D and calcium ingestion (1,200 mg per day) in daily food intake should be reinforced [3-5], and serum concentration of 25-hydroxyvitamin D testing is recommended for all patients with osteoporosis [4]. Supplementation is indicated for serum levels lower than 20 mg/dL [3], even though it does not seem to have a significant effect on BMD if persistent malnutrition and amenorrhea are present [4]. Despite the importance of estrogen function in reestablishing bone mass, sex hormone replacement with oral estrogen/progesterone treatment or testosterone supplementation has yet to show benefit in treating this pool of patients, possibly due to the multifactorial determinants of osteoporosis in AN [3-5].

Patients with osteopenia do not have an indication for a specific treatment, only general recommendations, such as weight gain (aiming for normal weight), adequate calcium and vitamin D ingestion (supplementation when needed), menstrual cycle regularization in female patients, and biannual monitoring with osteodensitometry [3,4]. In fact, some authors recommend osteodensitometry in patients with food disorders and amenorrhea lasting around 12 months [4].

In the adolescent population, the typical pharmacological measures used in adulthood for treatment or prevention of osteoporosis are not recommended, being weight gain and regulation of gonadal function, the most effective and available strategies in this specific group [2,5]. Physical activity is overall beneficial for bone health; however, in patients with AN, it can be excessive and become a compulsion, observed in up to 80% of this population, thus possibly having a paradoxical effect [1,5]. Patients with significantly reduced BMD must be warned against the practice of dangerous sports due to the risk of osteoporotic fractures [4].

The literature describes that AN can triplicate the risk of occurring fractures throughout life, with 57% of women having at least one once in a lifetime [1,5]. Additionally, the occurrence of long bone and vertebrae fractures frequently results in a decrease in height and chronic back pain, culminating in greater morbimortality and healthcare costs [1,3].

## Conclusions

This case aims to highlight the close relationship between restrictive food disorders and the development of complications, specifically the decrease of BMD and subsequent development of osteoporosis and its related fractures. In retrospective analysis and particularly in this case, given the absence of traumatic history, this diagnosis would have required an early high level of suspicion. Despite the recent diagnosis of AN, the patient had already begun caloric restriction practices for over three years, putting her at a higher risk of decreased BMD and, thus, osteoporosis. Although rare in young and healthy people, when associated with restrictive food disorders, it should raise a red flag in its clinical evaluation.

Preventing osteoporosis development and reduction of fracture risk in this population is essential. The current absence of consistent evidence regarding screening of osteoporosis in this particular group, greatly due to the lack of an effective method, should raise awareness and promote further larger-scale studies to establish standardized recommendations concerning not only screening but also pharmacological treatment of osteoporosis in patients with AN.

## References

[1] Steinman J, Shibli-Rahhal A (2019). Anorexia nervosa and osteoporosis: pathophysiology and treatment. J Bone Metab.

[2] Chidiac CW (2019). An update on the medical consequences of anorexia nervosa. Curr Opin Pediatr.

[3] Mehler PS (2019). Clinical guidance on osteoporosis and eating disorders: the NEDA continuing education series. Eat Disord.

[4] Jagielska GW, Przedlacki J, Bartoszewicz Z, Racicka E (2016). Bone mineralization disorders as a complication of anorexia nervosa - etiology, prevalence, course and treatment. Psychiatr Pol.

[5] Hung C, Muñoz M, Shibli-Rahhal A (2022). Anorexia nervosa and osteoporosis. Calcif Tissue Int.

